# Exploring Pan-Genomes: An Overview of Resources and Tools for Unraveling Structure, Function, and Evolution of Crop Genes and Genomes

**DOI:** 10.3390/biom13091403

**Published:** 2023-09-17

**Authors:** Sushma Naithani, Cecilia H. Deng, Sunil Kumar Sahu, Pankaj Jaiswal

**Affiliations:** 1 Department of Botany and Plant Pathology, Oregon State University, Corvallis, OR 97331, USA; pankaj.jaiswal@oregonstate.edu; 2 Molecular & Digital Breeing Group, New Cultivar Innovation, The New Zealand Institute for Plant and Food Research Limited, Private Bag 92169, Auckland 1142, New Zealand; cecilia.deng@plantandfood.co.nz; 3 State Key Laboratory of Agricultural Genomics, Key Laboratory of Genomics, Ministry of Agriculture, BGI Research, Shenzhen 518083, China; sunilkumarsahu@genomics.cn

**Keywords:** pan-genomes, comparative genomics, plant pathways, gene annotation, gene ontology, gravitropism

## Abstract

The availability of multiple sequenced genomes from a single species made it possible to explore intra- and inter-specific genomic comparisons at higher resolution and build clade-specific pan-genomes of several crops. The pan-genomes of crops constructed from various cultivars, accessions, landraces, and wild ancestral species represent a compendium of genes and structural variations and allow researchers to search for the novel genes and alleles that were inadvertently lost in domesticated crops during the historical process of crop domestication or in the process of extensive plant breeding. Fortunately, many valuable genes and alleles associated with desirable traits like disease resistance, abiotic stress tolerance, plant architecture, and nutrition qualities exist in landraces, ancestral species, and crop wild relatives. The novel genes from the wild ancestors and landraces can be introduced back to high-yielding varieties of modern crops by implementing classical plant breeding, genomic selection, and transgenic/gene editing approaches. Thus, pan-genomic represents a great leap in plant research and offers new avenues for targeted breeding to mitigate the impact of global climate change. Here, we summarize the tools used for pan-genome assembly and annotations, web-portals hosting plant pan-genomes, etc. Furthermore, we highlight a few discoveries made in crops using the pan-genomic approach and future potential of this emerging field of study.

## 1. Introduction

In recent years, advancements in affordable sequencing platforms and computational resources have helped to generate reference-quality genome assemblies for multiple crop accessions/varieties belonging to a single species. Thus, crop genomics has transitioned to the pan-genomic era. This shift has been made possible by advances in sequencing technology (i.e., short-read Illumina sequencing, long-read PacBio sequencing with low errors, Nanopore sequencing) and bioinformatics tools employed for genomic data processing and genome assemblies (for excellent reviews, see [1,2,3,4,5,6,7]). Whole-genome assembly-based pan-genomes have been reported for a few plant species, including rice [8,9,10], barley [11], wheat [12], maize [13], soybean [14], a wild relative of soybean *Glycine soja* [15], and brassica [16]. In particular, PacBio HiFi reads proved very useful for high-quality polyploid genome assemblies for peanut [17], wheat [18], oilseed [16], strawberry [19], and potato [20]. 

**Figure 1:**
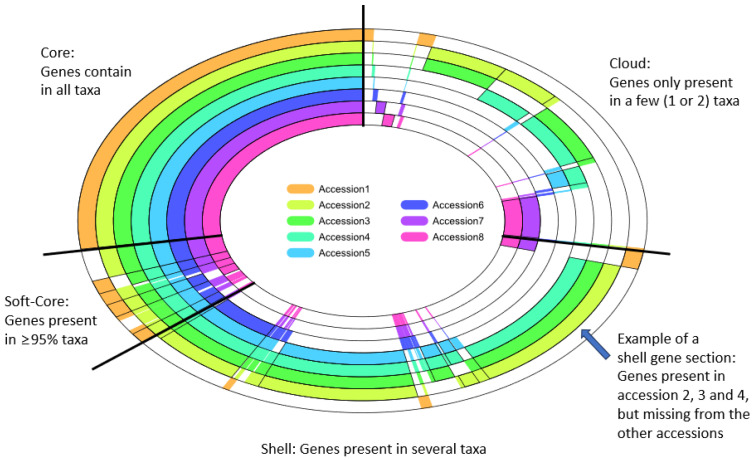
A conceptual depiction of the pan-genes across genomes of eight accessions belonging to the same clade. Each ring represents one accession. The top-left “core genes” represent conserved genes across eight accessions. The white section in a ring indicates the absence of ortholog(s). The “soft cores” represent genes found in ≥95% of accessions. The “cloud genes” are found only in one or two taxa. The rest between the “cloud genes” and “soft core genes” are “shell genes”.

The intraspecies genome comparisons of crops suggest extensive structural variation across diverse genotypes that affect both the genomic contents and plant function [14,21,22,23]. The structural variations among genotypes of the same species include insertion deletions (indels) and translocation of the small or large genomic regions that further cause presence–absence variations, copy number variations, chromosomal rearrangements, and variations in repeat sequences (i.e., tandem gene duplications, repetitive sequences in non-coding regions of the genome, transposable elements, centromere repeats, etc.). The conserved genes present in all cultivars/genotypes/subspecies/strains within a species constitute the “core” genome, and the variable genes represent the “dispensable” or “accessory” genome [2,24]. As shown in Figure 1, the accessory genome consists of the “shell genes” (found in most cultivars within a species) and the “cloud genes“ (present in only a small fraction of cultivars of the same species). 

Thus, a single reference genome represents only a fraction of the species-wide genomic space, and a pan-genome represents species-wide genomic space [2,13,15,25,26]. Often, the pan-genomes encompass genes found in crop wild relatives and ancestral species [27,28,29,30,31]. We would like to note here that many useful genes lost during crop domestication and extensive plant breeding [32,33,34] may be found in the “dispensable”/accessory genome of any crop species [15,33,35,36,37,38,39]. Thus, the availability of plant pan-genomes allows researchers and breeders to explore important candidate genes for improving crop yield, nutritional quality, and adaptability to changing climatic conditions and diseases. For instance, a few comparative genomic studies have revealed that gene amplification plays a vital role in disease resistance, abiotic stress tolerance, and other agronomic traits associated with plant development, architecture, and yield [40,41,42,43,44,45,46,47,48,49]. In addition, the high-quality pan-genomes also make it possible to study previously inaccessible regions of the eukaryotic genomes, including centromeres, long heterochromatic blocks, rDNA regions, etc., that exhibit low recombination, and provide new insights into crop genome evolution [50].

Recently, many excellent reviews have been published on plant pan-genomes [1,2,51,52,53,54], which focus on pan-genome construction, structure variation detection, challenges associated with polyploid crops, and the application of pan-genomes in crop research. It is important to highlight that none of the published reviews on pan-genomes provide a comprehensive collection of pan-genome tools or resources accessible in the public domain. Thus, our work fills this gap by concentrating on the current landscape of available pan-genome tools and resources tailored to the needs of crop researchers. Here, we review the current tools used for constructing and visualizing crop pan-genome data, public genomic portals/resources hosting pan-genes, pan-genome data, and pan-genome browsers. Furthermore, we highlight a few studies that have exploited a pan-genomic approach for discovering candidate genes associated with important agronomic traits. We also discuss the potential of pan-genome-driven translational research.

## 2. Pan-Genome Construction, Visualization, and Data Analysis Tools

The first step in setting up a pan-genome infrastructure is the selection of a diverse set of representative genotypes for sequence assembly that capture as many genetic variants as possible with a limited panel of genotypes [14,21,55,56]. The second step is the sequencing of individual genomes. The high-quality reference genomes are of critical importance for building pan-genome assemblies and a complete pan-gene atlas. Therefore, we see overlap and inter-connection between the genomics and pan-genomics. We envision pan-genomics as a natural extension and outgrowth of genomics, not a different field of study. The third step is the assembly and construction of the pan-genome. Previously a few reviews [1,57,58,59] have been published on several approaches implemented for pan-genome assembly. Here, we briefly describe the basic tenets of three popular methods. 

**Figure 2:**
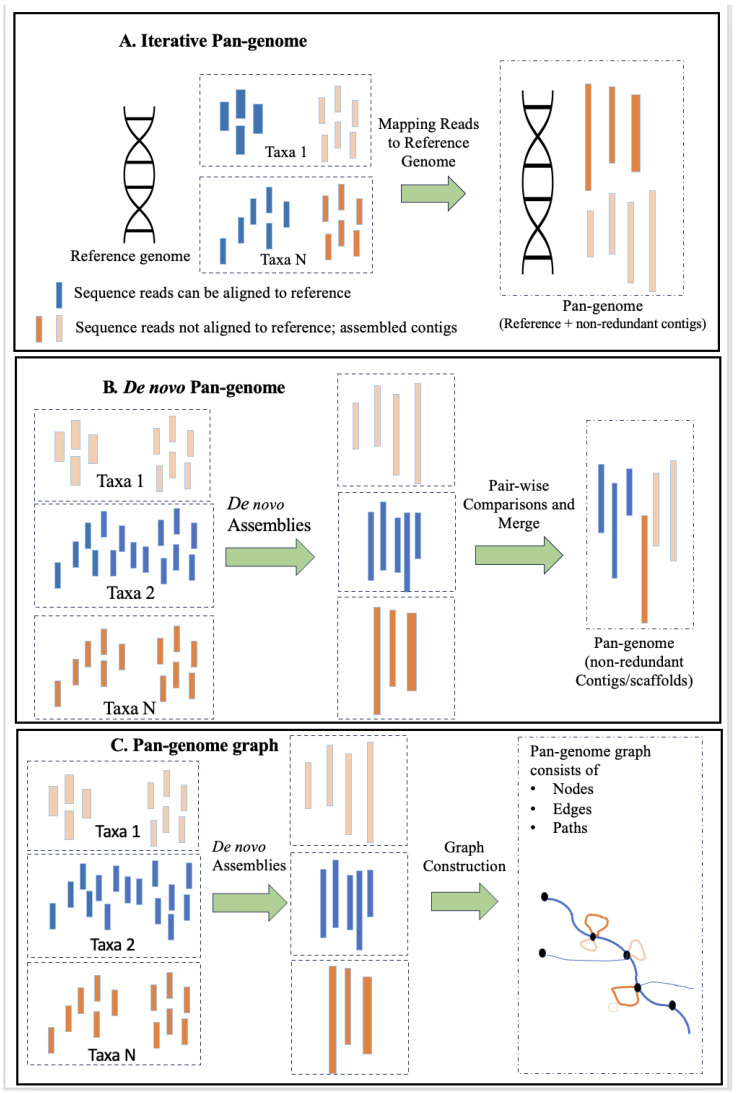
An illustration of three popular approaches currently used for pan-genome construction, including (**A**) reference-based iterative method, (**B**) de novo genome assembly, and (**C**) graph-based pan-genome assembly.

The first approach uses a high-quality reference genome for mapping sequence reads generated from all other genotypes. Iterative refinement allows for a progressive improvement of the assembly with additional data. This strategy can minimize errors by exploiting the information from a high-quality reference genome and limiting the coordinate consolidation issue (Figure 2A). However, this method requires the availability of a high-quality reference genome, which may not be available for all species or strains. Secondly, it is sensitive to misalignment errors or inaccuracies in the reference genome, which can potentially propagate errors throughout the assembly. Likewise, bias towards the reference genome may limit the detection of novel or divergent sequences. 

In species without access to a reference genome, de novo assembly of individual genotypes is generated, followed by mapping assembled contigs to each other [27]. The de novo genome assemblies have become a method of choice due to the advances in long-read sequencing and the availability of fast algorithms for aligning long-reads to call structural variants [7]. Conceptually, de novo assembly of multiple high-quality reference genomes and their comparison by pair-wise sequence alignment is arguably the most powerful and accurate approach to detect sequence variants from base-level resolution to novel genomic elements and rearrangements (Figure 2B). However, generating assemblies of polyploid plant genomes is still challenging, as current methods are limited in detecting and phasing heterozygous structural variants that erroneously produce chimeric contigs joining different haplotypes or ignore alternative haplotypes [60,61]. This approach is time-intensive and requires significant computational and bioinformatic resources, especially for large genomes and complex variations. Repeat resolution can be challenging, leading to fragmented assemblies, as it relies heavily on sequencing depth to overcome repetitive regions and complex variations.

**Figure 3:**
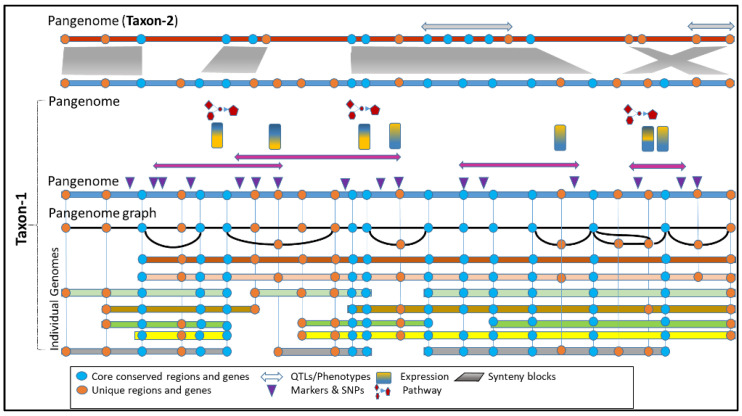
A conceptual view of a pan-genome reference graph carrying chromosomal rearrangements and mapped features. The graph allows views and analysis of whole-genome alignments, pan-gene sets, gene orthology, expression, pathways, function, and aligned synteny to help accelerate knowledge discovery and hypothesis-driven research.

The third graph-based approach allows adding any variant to the reference as a node at the genomic location where it is discovered, and then haplotypes are associated with one of the reference genomes used to build the graph. Reads are then realigned to this genome, leading to more accurate mapping. This method can accommodate new genomic data through iterative refinement, allowing for continuous improvement of the pan-genome assembly (Figure 2C). However, graph construction and traversal can be computationally intensive, especially for large and diverse pan-genomes, and require substantial computational resources. Typically, graph complexity increases with the addition of more genomes, potentially impacting scalability and computational efficiency. Nonetheless, the graph-based pan-genomes can represent complex variations, including structural variants and large-scale rearrangements, facilitating the identification of shared and unique genomic regions among individuals or strains and aiding in excellent visualization of pan-genomes. A conceptual visualization of the graph-based pan-genome is shown in Figure 3. 

Recently, Shang et al., 2022 [21] have constructed a ‘Super Pan-genome of rice’ containing high-quality assemblies of 251 rice genomes, including 202 accessions of domesticated Asian rice *Oryza sativa*, 28 accession of *Oryza rufipogon* (the wild ancestor of *O. sativa*), 11 accessions of domesticated African rice *Oryza glaberrima*, and 10 accessions of *Oryza barthii* (the wild ancestor of *O. glaberrima*). They used the de novo long-read assembly and a graph-based approach. The Rice Super Pan-genome Information Resource Database (RiceSuperPIRdb) provides access to a reference-free whole-genome multiple sequence alignment for these 251 rice accessions. This resource hosts fully annotated pan-genome graph visualization using the JBrowse genome browser. It facilitates the integration of structural variations, gene annotations, transposable element annotations, pan-genome graphs, and BLAST tools [21].

A few excellent reviews have previously described the development of computational tools for pan-genome visualizations [57,58,62,63]. We note here that genome sequencing technologies and assembly algorithms are rapidly evolving to achieve high accuracy complemented by additional independent mapping approaches, such as optical maps and Hi-C, to validate structural variant calls (i.e., inversions and translocations). It is important to acknowledge that the details and outcomes of each method may vary based on the specific pan-genome assembly tools, parameters, and characteristics of the genomic data employed in the process [64,65]. Here, we compiled a summary of the latest representative tools in Table 1. It is crucial to recognize that the development of pan-genome tools is an active field, and the list could not be exhaustive.

## 3. A Survey of Crop Pan-Genome Portals and Data Resources 

With technical advances and the affordability of the sequencing and assembly of genomes, we are experiencing a deluge of big data in biology. The plant research community now faces a bigger challenge of making genomic data findable, accessible, interoperable, and reusable (FAIR) [111,112]. Public databases and genomic resources play a crucial role in making genomic data FAIR and provide tools for analyses and visualization of genomic, transcriptomic, proteomic, and metabolomic data [113,114,115,116,117,118,119,120]. Furthermore, the secondary knowledgebases synthesize and curate knowledge graphs, providing information for gene–gene interactions, metabolic networks, and pathways, and providing the tools for analyses of user’s data in the context of plant genome browser or pathways [90,97,114,115,119,121,122,123,124,125,126,127,128,129,130]. Currently, a substantial number of plant genome browsers, amounting to a few hundred, can be accessed through platforms like Plant Ensembl [120], Phytozome [131], and various clade-specific community databases (for a recent review, see [132]). In particular, the pathway databases and species-specific metabolic networks curate data at the species level and, thus, can easily accommodate the knowledge gained from the genome analysis of multiple accessions belonging to the same species. If some of the critical genetic hotspots or genomic loci associated with metabolism or production of specific metabolites production are absent in the reference genome (or not annotated correctly), the pan-genome data can help pathway database biocurators to incorporate data from multiple representative genotypes and build the accurate representation of metabolome present in a species or clade. The availability of pan-genomes would reduce the occurrence of false negatives. However, the availability of plant pan-genome portals is limited and experiencing slow growth. To provide an overview of the current state of crop pan-genomic research, resources, and portals, we compiled Table 2.

**Figure 4:**
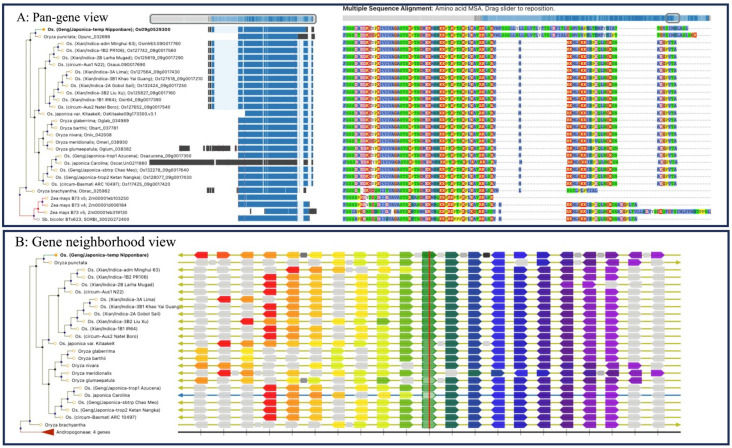
A pan-gene overview for TAC1 transcription factor (reference gene *OsTAC1*; Os09g0529300) and its orthologs from various accessions of cultivated rice *O. sativa,* other members of *Oryza* genus, and two other monocots maize and sorghum at Gramene oryza pansite. Users can explore (**A**) a multiple protein sequence alignment of TAC1 orthologs and (**B**) gene neighborhood conservation.

It is clear from Table 2 that crop pan-genome research is at its early stage. The pan-genome browsers are available for a few crops, and thus, most of the data is not supported for user-friendly query, visualization, and analysis of the user’s data. However, the few platforms and genomic databases that support pan-gene analysis in a phylogenomic context and support the user’s query show the potential of pan-genome data for supporting basic research as well as translational applications for crop improvement. Here, we highlight an example of visualizing the pan-gene data for the *TILLER ANGLE CONTROL 1* (*OsTAC1*) transcription factor coding gene from various accessions of cultivated rice *O. sativa* and other members of *Oryza* genus at Gramene (https://oryza.gramene.org; accessed on July 20, 2023). *OsTAC1* is induced by gravity stimulation and promotes horizontal shoot growth by negatively regulating shoot gravitropism [160]. Thus, it is involved in regulating tiller angle and modulating plant architectural traits of agronomic importance. A comparison of TAC1 protein sequences shows a significant variability at the carboxy-terminal between the domesticated rice cultivars of japonica and indica accessions (see Figure 4A). Indeed, a previously published study has shown that a point mutation in the *OsTAC1* gene at the 3′-splicing site of the 1.5-kb intron (‘GGGA’) in japonica rice accessions caused a reduction in the expression of this gene, leading to a smaller tiller angle. This trait was selected in the japonica rice accessions. In contrast, wild rice accessions and indica rice accessions with large tiller angles contain ‘AGGA’ sequences at the 3′-splicing site of the 1.5-kb intron [161]. The *OsTAC1* gene is on chromosome 9 in the rice genome and shows high conservation across all rice accessions (Figure 4B). However, we also see that the *OsTAC1* gene neighborhood on the left-hand side is not much conserved, and it remains to be explored for candidate genes involved in the functional adaptation of rice accessions.

In addition to the Gramene database, a few more pan-genome portals provide a similar view of pan-genes visualization and analysis tools. For example, the Banana Genome Hub uses the Panache platform to visualize pan-genome data on *Musaceae*. However, the well-established crop genome portals generally support users in exploring genes and gene families, chromosome structures, synteny, structural variations, gene expression patterns, SNP markers, etc.

## 4. Plant Pan-Genomics-Driven Insights for Understanding the Basis of Agronomic Traits

Cereal crops have been the prime subject of agriculture research. Thus, we have matured genomic resources, enriched genome annotations, and genotype and sequence data facilitating comparative genomics and pan-genomics studies (See Table 2). The focus of all categories of genomic research on cereal crops aims to increase the grain yield or plant developmental, physiological, and architectural traits that can support the higher yield. For example, Wang et al. (2022) [109] used rice pan-genome to identify *GW5* genes associated with the trait ‘thousand-grain weight’ (TGW) and a novel locus *qPH8-1* involved in the regulation of plant height. In another study by Shang et al., 2022 [21], a super pan-genome of rice was constructed helped to identify genetic variants associated with submergence tolerance, seed shattering, and plant architecture [21]. Many important studies have been published on maize, rice, and wheat, and discoveries are being implemented for their improvement. 

Notably, more recently, investments are being made in the genomic and pan-genomic research of minor cereal crops, including sorghum and millets, suitable for growing in diverse and marginal lands (see Table 2). These crops have a high degree of in-built tolerance for mitigating the impact of harsh environments, and resistance against many pests and pathogens. For example, foxtail millet (*Setaria italica*) is a model plant for studying C4 photosynthesis and developing climate-resilient crops. A pan-genomic study of foxtail millet identified an important genetic variation in the promoter region of *SiGW3* that is associated with yield improvements [22]. Another study identified 13 marker-trait associations using proso millet (*Panicum miliaceum* L.) pan-genome [162]. Similarly, Yan et al. (2023) recently constructed a pearl millet pan-genome that helped to identify over 400,000 genomic structural variants and provided insights into heat tolerance. This study also identified the RWP-RK gene conferring enhanced heat tolerance [55]. Another group of previously understudied crops is legumes that have gained from genomic and pan-genomic research and breeding efforts (see Table 2). For example, pigeon pea is an important orphan crop mainly grown by smallholder farmers in the tropics and subtropical regions of the world. It has an in-built tolerance for drought stress and is very productive in marginal land with small inputs. A pan-genome study of pigeon peas identified 225 SNPs associated with nine agronomically important traits. These associations will aid pigeon pea germplasm improvement [154]. In another study, Liu et al. (2022) analyzed 217 mung bean accessions and discovered many novel genes associated with agronomic traits, including an SNP in the candidate genes SWEET10 homolog (jg24043) associated with crude starch content; NRT1/PTR FAMILY 2.13 gene for pod length; a homolog of WUSCHEL-family homeobox gene associated with yield; and a gene presence-absence variation in a multi-gene locus associated with color-related traits [163]. Mung bean is an excellent plant-based source of protein and is grown in temperate, subtropical, and tropical regions.

Pan-genome studies have become increasingly important for understanding the genetic diversity of major crops from tropical regions, including cassava (*Manihot esculenta*) and banana (*Musa* spp.). These crops are a vital component of diverse ecosystems and play essential roles in the livelihoods of local communities. Insights gained from genomic and pan-genome research are being used for improving banana resistance against Fusarium wilt to safeguard the global banana industry [99,164]. Similarly, the pan-genome of cassava (*Manihot esculenta*), a staple crop for millions of people in Africa, is helping to score genomic variations, particularly in genes associated with disease resistance and starch biosynthesis [151]. More studies are being performed on important fruits and vegetables (i.e., banana, apple, tomato, melon, citrus, and grape) and oilseed crops (i.e., Brassica, soybean, sunflower, etc.) listed in Table 2. A few studies have uncovered new genes and rare alleles that regulate secondary metabolites associated with color and flavor [46,49,165], pathogen resistance [43,47,56,79,145,165,166,167], and abiotic stress tolerance [41,42,55]. These findings foster an understanding of the genetic basis of diverse traits in domesticated crops and offer promising prospects for introducing candidate genes (for disease resistance and quality traits) through molecular breeding or precise genome editing into elite cultivars. 

**Figure 5:**
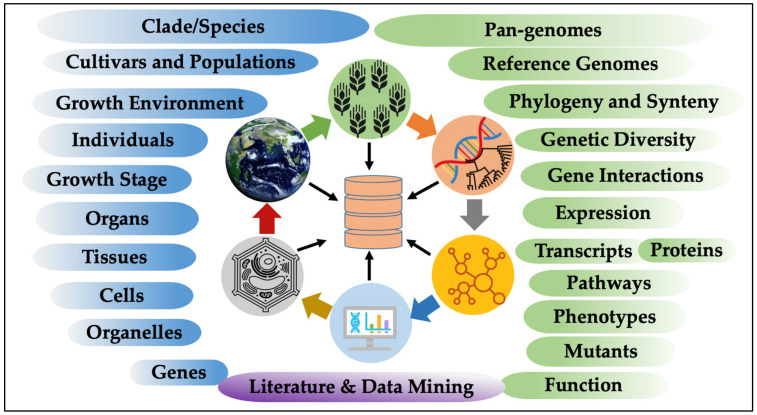
Plant pan-genome browsers can help to integrate heterogeneous omics data to understand gene function, genome evolution and speciation; to establish genotype to phenotype connections; and enable genomic selection, genome editing, and phenotype prediction to support and sustain agriculture production.

In conclusion, the acquisition of pan-genomes holds immense potential for pursuing fundamental questions related to the evolution of crop genomes as well as for breeding high-yielding crops that are resilient to a range of biotic and abiotic stresses. Utilizing pan-genomes enables researchers to (1) identify gains and losses of genetic regions and structural variations strongly associated with desirable fitness phenotypes such as abiotic stress and disease tolerance, growth and development, yield, biomass, and performance. (2) Identify gains and losses in protein-coding regions and/or epigenetic features between crops and closely related species. (3) Identify pathways, gene networks, expression profiles, and transcript isoforms that correlate to given major and minor quantitative trait loci (QTLs) with desired phenotypes and adaptation traits. (4) Project functional and phenotype homologs from a well-studied species onto a new/less-studied species through whole genome comparisons and synteny. (5) Advance plant breeding efforts by mapping/querying/visualizing public or personal project data to build or test hypotheses, discover markers, and gain knowledge. In summary, harnessing the full potential of pan-genomes for targeted crop breeding is an important goal of the plant research and breeding community. The integration of diverse genomic data and their visualization in the context of pan-genome is crucial for supporting marker-assisted selection, genomic selection, and gene editing efforts towards developing crops that can adapt and thrive in a range of climates and environments, securing global food security and sustainability (see Figure 5). 

## 5. Outlook, Opportunities, and Innovations in Plant Pan-Genome Research

Plant genomes are often very large and complex, making it difficult to produce high-quality genome assemblies with accurate gene annotations. For over last three decades, genotypic variations have been captured using various genetic markers, including RFLP, RAPD, SNPs, SSRs, microsatellite markers, etc., to establish connections between genotype and phenotype to aid plant breeding and cultivar improvement [168,169,170,171,172,173]. However, the advances in the next-generation sequencing and computational methods required for processing large-scale genomic and transcriptomic data have facilitated rapid and cost-effective whole-genome sequencing [7,174,175] and are now driving pan-genomic research. For the first time in history, researchers are able to explore intra- and inter-species structural variations at the resolution of nucleotide sequence level. We expect a significant expansion in pan-genomes availability across a broader range of plant species. The pan-genomes facilitate the identification of genes conserved across species and genes unique to specific species or a subset of accessions. The availability of species-specific or genus-level pan-genomes of crops is crucial for understanding the dynamics of their genome evolution, including the impact of artificial selection and domestication, crop diversification, and adaptation under varied environments [50,174,176,177]. For instance, a super-pan-genome of the *Citrullus* genus comprising 346 cultivated watermelon accessions and 201 wild accessions suggested that a duplication of the sugar transporter gene *ClTST2* was likely selected during domestication for higher fruit sweetness, and the wild accessions harbor many genes related to disease resistance [178].

In addition, plant pan-genomes can help to advance the fundamental understanding of the plant kingdom at various scales (see Figure 5). It can aid in improving functional annotations of genes and genomes [179,180] and provide insights into specific roles and interactions of different genetic elements. Furthermore, pan-genomes can help in understanding the evolution of metabolic diversity across diverse taxonomic clades [97]. This knowledge has implications for improving crop traits and developing more resilient and sustainable agricultural practices. Moreover, by analyzing the genomic diversity represented in pan-genomes, scientists can understand the distribution and composition of vegetation across different environments. This information is critical for conservation efforts, ecological studies, and developing strategies to protect and sustainably manage plant resources. 

Finally, we see a great application of pan-genome data in improving gene annotations and identifying evolutionary conserved sets of genes associated with important agronomic traits. Likewise, comparative genomic studies can help to identify and annotate clade-specific unique genes that determine metabolite compositions of important fruit and vegetable crops or other categories. These specialized metabolic pathways and associated entities can be easily curated in the pathway databases [97,121,123,124,128,181,182]. The workflows for interspecies gene family comparisons, GO annotations, and standard protocols for gene biocuration are very efficient and established; they can easily accommodate the insights gained from intra-species comparisons. For instance, the availability of whole genome sequences of plants has contributed tremendously to the knowledge of gene duplications, gene family evolution, and functional diversification of homologous genes [179,180,183]. Gene Ontology (GO) and Plant Ontology (PO) annotations have played a central role in accessing the potential gene functions [184,185,186]. Furthermore, the comprehensive analysis of plant transcriptomes has helped us to link genes with potential biological processes, pathways, and responses to biotic and abiotic stress or stimulants [179,180,187,188,189]. Integrating pan-genomes with other omics, such as transcriptomics, epigenomics, proteomics, and phenomics data, will enable a comprehensive understanding of gene regulation and functional mechanisms underlying important agronomic traits. In conclusion, as shown in Figure 5, pan-genomic research drives functional genomics, evolutionary studies, and biodiversity exploration and holds great potential for crop improvement, environmental conservation, and sustainable agriculture. 

It is important to emphasize that pan-genome construction and whole genome-level comparative analysis require substantial computational infrastructure and expertise in various facets, including sequence generation, genome assembly and annotation, and subsequent bioinformatic analyses and data visualization. In general, all these tasks are beyond the capacity of individual research laboratories, and thus, they require extensive infrastructure and bioinformatic support from their institutions and public databases. Public data repositories, databases, genomic resources, and secondary knowledgebases play an essential role in aiding the community of researchers in providing ontologies [184], archiving and annotating genomic data, and supporting analysis and visualization of omics data to support data-driven hypotheses and making experimental plans [90,97,115,128]. Here, we have reviewed the resources and tools available to the plant research community for pan-genomic research.

We note here that the resources and tools to support researchers in exploring pan-genomic data are limited and at an early developmental stage. We have witnessed a growing number of publications on crop pan-genomes in the last five years (see Table 2); however, the pan-genomic and genomic diversity data for the majority of the crops are stored and archived with no associated tools/features required for visualization and effective use by other researchers. Thus, advancement and innovations in pan-genomic data visualization, analysis tools, and additional biocuration of genomic data are needed to facilitate meaningful intraspecies and interspecies genomic comparisons. Community biocuration plays an essential role in making sense of the big data and ensuring quality controls at various steps [190]. It could allow researchers to study pan-genomes more thoroughly and identify genes and genomic regions associated with important traits. Equally important is integrating crop pan-genomes in comprehensive knowledgebases that host analyzed and annotated genomic and pathway data for ensuring the FAIR data policy implementations [111,132].

From a technical aspect, integrating machine learning and artificial intelligence techniques will expedite the analysis of complex pan-genomic datasets, aid in identifying patterns, and accelerate the construction of predictive models and gene biocuration. Furthermore, the genomic resources are more mature for the model plants and major crop species [132]. However, the necessary financial and infrastructure support for minor crops, fruit and vegetable crops, and orphan crops is still insufficient [191,192,193]. Although the cost of sequencing a single genome has decreased significantly in recent years, sequencing multiple genomes can still be prohibitive, a significant barrier to conducting pan-genome research in poorly studied crops or orphaned crops. We expect that this review will help researchers find appropriate tools and resources relevant to pan-genome construction and analysis. Secondly, it will help to understand and evaluate the strategies employed for pan-genomic studies in crops and encourage them to seek necessary collaborations within the community. We take this opportunity to advocate for increased funding for developing the infrastructure, tools, and biocuration of genomic data. Furthermore, we hope this review can help students and young researchers learn about the status and the future potential of pan-genomics.

## Figures and Tables

**Table 1:** A list of popular open-source tools for pan-genome assembly and visualization. All URLs were checked and confirmed to be valid on 13 September 2023.

Tool Name and URL	Remarks and Citation
Genome assembly	
Hifiasm https://github.com/chhylp123/hifiasm (accessed on 13 September 2023).	Constructs haplotype-resolved assemblies from accurate HiFi Reads [60].
Canu https://github.com/marbl/canu (accessed on 13 September 2023).	Assembles genomes of any size from single molecule sequences and provides graphical fragment assembly that can be integrated with complementary phasing and scaffolding methods [66].
Flye https://github.com/fenderglass/Flye (accessed on 13 September 2023).	Assembles single molecule, long-read sequencing data into genomes using repeat graphs [67].
PAGIT https://www.sanger.ac.uk/tool/pagit (accessed on 13 September 2023).	PAGIT is a package of tools for generating high-quality draft genome sequences by ordering contigs, closing gaps, correcting sequence errors, and transferring annotation. PAGIT is compiled for Linux/UNIX systems and is available as a virtual machine [68].
MEGAHIT https://github.com/voutcn/megahit (accessed on 13 September 2023).	Ultra-fast NGS assembler for metagenomes [69].
SPADes https://cab.spbu.ru/software/spades/ (accessed on 13 September 2023).	A set of genome assembly and analysis tools that can use long- and short-read sequence data [70,71].
Pan-genome graph construction, normalization, identification of structural variants, and visualization	
Vgtools: vg construct, vg call, vg giraffe, vg map or vg mpmap https://github.com/vgteam/vg (accessed on 13 September 2023).	Toolset for eukaryotic pan-genome graph construction, read mapping, variant calling, and graph visualization [58].
Minigraph https://github.com/lh3/minigraph (accessed on 13 September 2023).	Tool for graph construction, mapping, and variant calling [72].
ODGI https://github.com/pangenome/odgi (accessed on 13 September 2023).	Optimized Dynamic Genome/Graph Implementation (ODGI) is a tool suite representing graphs, including structurally complex regions, with minimal memory overhead [73]. It is a pan-genome toolbox with more than 30 tools to transform, analyze, simplify, validate, annotate, and visualize pan-genome graphs.
PGGB https://github.com/pangenome/pggb (accessed on 13 September 2023).	Uses ODGI as the backbone for pan-genome graph construction, normalization, and visualization [74].
MGRgraph https://github.com/LeilyR/Multi-genome-Reference (accessed on 13 September 2023).	An algorithm for building a multi-genome graph.
Cactus https://github.com/ComparativeGenomicsToolkit/cactus (accessed on 13 September 2023).	A reference-free multiple genome alignment program that can use progressive mode to build pan-genome across different species [75,76]
PanTools https://pantools.readthedocs.io/en/latest/user_guide/install.html (accessed on 13 September 2023).	A platform for pan-genome graph construction, read mapping, phylogeny analysis, pan-graph query, and pan-gene annotation [77].
Smoothxg https://github.com/pangenome/smoothxg (accessed on 13 September 2023).	A tool for local reconstruction of variation graphs.
nf-core/pangenome https://github.com/nf-core/pangenome (accessed on 13 September 2023).	Nextflow pipeline for all-vs-all alignment, pan-genome graph construction, normalization, remove redundancy, and visualization (through ODGI) [78].
SeqWish https://github.com/ekg/seqwish (accessed on 13 September 2023).	Builds a variation graph from pairwise alignments [73].
PanPipe https://github.com/USDA-ARS-GBRU/PanPipes (accessed on 13 September 2023).	An end-to-end pan-genome graph construction and genetic analysis pipeline [79].
PanGene https://github.com/lh3/pangene (accessed on 13 September 2023).	Used for ortholog and paralog analysis and for building pan-gene graphs.
Minimap2 https://github.com/lh3/minimap2 (accessed on 13 September 2023).	A fast DNA or long mRNA sequence aligner to a reference genome [80].
NGMLR https://github.com/philres/ngmlr (accessed on 13 September 2023).	This program aligns PacBio long reads to genomes for detecting complex structural variations [5].
MUMmer4 https://mummer.sourceforge.net/ (accessed on 13 September 2023). https://github.com/mummer4/mummer (accessed on 13 September 2023).	A genome-to-genome aligner tool [81].
GraphAligner https://github.com/maickrau/GraphAligner (accessed on 13 September 2023).	A tool for aligning long reads to genome graphs [82].
V-ALIGN https://github.com/tcsatc/V-ALIGN (accessed on 13 September 2023).	V-ALIGN allows gapped sequence alignment directly on the input graph and supports affine and linear gaps [83].
PaSGAL https://github.com/ParBLiSS/PaSGAL (accessed on 13 September 2023).	Parallel Sequence to Graph Aligner (PaSGAL) facilitates local sequence alignment of sequences to variation graphs, splicing graphs, etc.
GED-MAP https://github.com/thomas-buechler-ulm/gedmap (accessed on 13 September 2023).	A tool for mapping short-read sequence data to the pan-genome graph [84].
DeepVariant https://github.com/google/deepvariant (accessed on 13 September 2023).	A deep learning-based variant caller that uses sequence read alignments in BAM and CRAM format to produce image tensors and convolutional neural networks to identify universal SNP and small-indel variants [85,86].
SpeedSeq https://github.com/hall-lab/speedseq (accessed on 13 September 2023).	A platform for alignment, variant calling, and functional annotation [87].
graphTyper https://github.com/DecodeGenetics/graphtyper (accessed on 13 September 2023).	This graph-based variant caller realigns short-read sequence data to a pan-genome for discovering sequence variants [88].
PanGenie https://github.com/eblerjana/pangenie (accessed on 13 September 2023).	An alignment-free Kmer-based genotyper for structural variation detection on pan-genome graphs. It uses short-read sequencing data to genotype a broad spectrum of genetic variation [89].
VEP https://ensembl.gramene.org/tools.html (accessed on 13 September 2023).	The Variant Effect Prediction (VEP) tool helps in analyzing the consequences of sequence variations on transcript structure and gene function [90].
OrthoFinder https://github.com/davidemms/OrthoFinder (accessed on 13 September 2023).	This method is used for finding orthologs in proteomes [91].
OrthoMCL https://orthomcl.org/orthomcl/app (accessed on 13 September 2023).	A scalable method for constructing orthology groups from eukaryotic proteomes [92].
JustOrthologs https://github.com/ridgelab/JustOrthologs/ (accessed on 13 September 2023).	JustOrthologs is a fast ortholog identification algorithm that uses the conservation of gene structure [93].
PhyloMCL https://sourceforge.net/projects/phylomcl/files/Materials/ (accessed on 13 September 2023).	PhyloMCL provides accurate clustering of hierarchical orthogroups guided by phylogenetic relationships and inference of polyploidy events [94].
OMA https://github.com/DessimozLab/OmaStandalone/tree/v2.4.0 (accessed on 13 September 2023). https://omabrowser.org/oma/home/ (accessed on 13 September 2023).	Orthologous Matrix (OMA) is a method for ortholog identification from genomes [95].
InParanoid-Diamond https://bitbucket.org/sonnhammergroup/inparanoid/src (accessed on 13 September 2023).	The tool is used for the identification of gene-orthologs and gene family clustering [96]. This is used for orthology projection in the Plant Reactome (https://plantreactome.gramene.org) [97].
Panache https://github.com/SouthGreenPlatform/panache (accessed on 13 September 2023).	A web-based tool for viewing linearized pan-genomes [98]. For example, the banana genome hub [99].
MoMI-G https://github.com/MoMI-G/MoMI-G/ (accessed on 13 September 2023).	Genome graph browser for viewing structural variations. Users can filter and visualize annotations and inspect read alignments over the genome graph [100].
panGraphViewer https://github.com/TF-Chan-Lab/panGraphViewer (accessed on 13 September 2023).	panGraphViewer, based on Python3, is used for pan-genome graph visualization and runs on all major operating systems.
Bandage https://rrwick.github.io/Bandage/ (accessed on 13 September 2023).	An interactive tool for visualizing de novo assembled genomes [101].
Bandage-NG https://github.com/asl/BandageNG (accessed on 13 September 2023).	GUI program to interact with assembly graphs based on the Open Graph Drawing Framework (OGDF) and Open Graph Algorithms and Data Structures Framework).
sequenceTubeMaps https://github.com/vgteam/sequenceTubeMap (accessed on 13 September 2023).	Interactive visualization of genomes [102].
GfaViz https://github.com/ggonnella/gfaviz (accessed on 13 September 2023).	Interactive visualization of Graphical Fragment Assembly (GFA) genome graphs [103].
AGB https://github.com/almiheenko/AGB (accessed on 13 September 2023).	Assembly Graph Browser (AGB) is used for constructing and visualizing large assembly graphs and repeat sequence analysis [104].
IGGE https://github.com/immersivegraphgenomeexplorer/IGGE (accessed on 13 September 2023).	An interactive graph genomes browser.
GFAViewer https://lh3.github.io/gfatools/ (accessed on 13 September 2023).	Used for online visualization of GFA files.
SGTK https://github.com/olga24912/SGTK (accessed on 13 September 2023).	The scaffold graph toolkit is used for the construction and interactive visualization of scaffold graphs using sequencing data [105].
Maffer https://github.com/pangenome/maffer (accessed on 13 September 2023).	It converts sorted graphs to multiple alignment format (MAF).
Gfatools https://github.com/lh3/gfatools (accessed on 13 September 2023).	A set of tools to parse, subgraph, and convert GFA or rGFA format to FASTA/BED format.
Pgge https://github.com/pangenome/pgge (accessed on 13 September 2023).	It is a pan-genome graph evaluator
WGT https://github.com/Kuanhao-Chao/Wheeler_Graph_Toolkit (accessed on 13 September 2023).	This package contains tools and algorithms for recognizing, visualizing, and generating Wheeler graphs.
GBWT https://github.com/jltsiren/gbwt (accessed on 13 September 2023).	A tool used for haplotype matching and storage using the positional Burrows-Wheeler Transform (PBWT) approach [106,107].
Spodgi https://github.com/pangenome/spodgi (accessed on 13 September 2023).	Convert ODGI genome graph file to SPARQL database.
GraphPeakCaller https://github.com/uio-bmi/graph_peak_caller (accessed on 13 September 2023).	A tool for calling transcription factor peaks on graph-based reference genomes using ChIP-seq data [108].
PSVCP https://github.com/wjian8/psvcp_v1.01 (accessed on 13 September 2023).	It is a pan-genome analysis pipeline (PSVCP) to construct a pan-genome, call structural variants, and run population genotyping. It was used for rice pan-genome [109].
ppsPCP http://cbi.hzau.edu.cn/ppsPCP/ (accessed on 13 September 2023).	It is designed specifically for constructing fully annotated plant pan-genomes. It scans presence/absence variants [110].

**Table 2:** A list of pan-genome portals and data resources for crops. All URLs were checked and confirmed to be valid on 13 September 2023.

Pan-Genome Resource	Remarks
Gramene Link: https://www.gramene.org/pansites (accessed on 13 September 2023). Species: maize, rice, grapevine, and sorghum.	Gramene hosts 128 reference plant genomes [115] and pan-genome sites for maize, rice, grapevine, and sorghum.
SorghumBase Link: https://www.sorghumbase.org (accessed on 13 September 2023). Species: sorghum.	SorghumBase portal hosts a sorghum pan-genome browser comprising five sorghum reference genome assemblies and genetic variant information for natural diversity panels and ethyl methanesulfonate (EMS)-induced mutant populations [118].
RPAN Link: https://cgm.sjtu.edu.cn/3kricedb (accessed on 13 September 2023). In addition to RPAN, the data and analyzed outputs from 3K RGP are available at the following websites: http://snp-seek.irri.org/ (accessed on 13 September 2023). http://www.rmbreeding.cn/index.php (accessed on 13 September 2023). http://www.ricecloud.org (accessed on 13 September 2023). https://aws.amazon.com/public-data-sets/3000-rice-genome (accessed on 13 September 2023). Species: rice (*O. sativa*) and its wild relatives.	The Rice Pan-genome Browser (RPAN) hosts genomic variation data from 3010 diverse rice accessions [8,9,133,134]. It contains ~370 Mbp IRGSP genome and ~260 Mbp novel sequences comprising 50,995 genes (23,914 core genes). RPAN provides a phylogenetic tree browser to view the phylogeny of rice accessions and a genome browser to view gene annotation and presence-absence variations. Users can access pan-gene views and associated genetic variations.
RiceSuperPIRdb Link: http://www.ricesuperpir.com (accessed on 13 September 2023). Species: 251 genomes representing domesticated rice accessions and wild relatives (202 *O. sativa*, 28 *O. rufipogan*, 11 *O. glaberrima*, and 10 *O. barthii* accessions).	The RiceSuperPIRdb hosts a genome browser for the rice super pan-genome built using reference-free, high-quality whole genome alignment of 251 independent genome assemblies. Genome annotations and node-specific K-mer spectrum pan-genome graphs are available for each assembly. In addition, genetic variation graphs support linking query data and the identification of lineage-specific haplotypes for trait-associated genes [21].
PanOryza Link: https://panoryza.org (accessed on 13 September 2023). Species: magic-16 rice accessions; see https://panoryza.org (accessed on 13 September 2023).	PanOryza provides consistency in the rice gene annotation across all rice varieties and the rice pan-genome browser supported by the JBrowse genome browser.
MaizeGDB Link: https://nam-genomes.org (accessed on 13 September 2023). Species: maize.	MaizeGDB hosts 48 maize genomes, including 26 high-quality PacBio genome assemblies of the Nested Associated Mapping (NAM) population founder lines. It allows users to connect genomes, gene models, expression, methylome, sequence variations, structural variations, transposable elements, etc., across the maize pan-genome supported by the Jbrowse browser [125].
ZEAMAP Link: www.zeamap.com (accessed on 13 September 2023). Species: maize.	The ZEAMAP database incorporates multiple annotated reference genomes, data from transcriptomes, open chromatin regions, chromatin interactions, high-quality genetic variants, phenotypes, metabolomics, genetic maps, population structures, and populational DNA methylation signals from maize inbred lines [135].
GreenPhylDB Link: https://www.greenphyl.org/cgi-bin/index.cgi (accessed on 13 September 2023). Species: 46 plant species and 19 pan-genomes, including rice, maize, banana, grape, and cacao. In addition, it hosts 27 reference genomes.	GreenPhylDB is part of the South Green Bioinformatics platform (https://www.southgreen.fr) [136]. It aids exploration of gene families and homologous relationships among plant genomes.
The Wheat Panache Web Portal Link: http://www.appliedbioinformatics.com.au/wheat_panache (accessed on 13 September 2023). Species: wheat.	This wheat pan-genome graph visualization is supported by the Panache tool. It allows users to explore structural variations across the selected wheat accessions [137].
GrainGenes Link: https://wheat.pw.usda.gov/GG3/pangenome (accessed on 13 September 2023). Species: wheat, barley, rye, oat.	GrainGenes hosts molecular and phenotype data for wheat, barley, rye, oat, etc., including several genome assemblies, genome browsers, and a *T. aestivum* (bread wheat) pan-genome [138].
Wheat Pan-genome Link: http://appliedbioinformatics.com.au/cgi-bin/gb2/gbrowse/WheatPan/ (accessed on 13 September 2023). Species: bread wheat (*Triticum aestivum*).	The wheat Pan-genome facilitates comparison of an improved reference for the Chinese Spring wheat genome with 18 wheat cultivars [139].
SGN Links: https://solgenomics.net (accessed on 13 September 2023). Subsites: http://solomics.agis.org.cn/tomato/tool/jbrowse_nav (accessed on 13 September 2023). https://solgenomics.net/projects/tgg (accessed on 13 September 2023). https://solgenomics.net/organism/Solanum_melongena/genome (accessed on 13 September 2023). Species: tomato, potato, petunia, and eggplant.	The Solanaceae Genomics Network (SGN) database hosts pan-genome data for tomato and eggplant. International Tomato Genome Sequencing Project produced the tomato pan-genome data consisting of genome assemblies from 46 accessions (22 *Solanum lycopersicum*, 13 *Solanum lycopersicum* var. cerasiforme; and 11 *Solanum pimpinellifolium*) [140]. For details about the eggplant pan-genome and pan-plastome data, see Barchi et al., 2021 [141].
PepperPan Link: http://www.pepperpan.org:8012/ (accessed on 13 September 2023). Species: *Capsicum annuum* (pepper) and its wild relatives.	The PepperPan was constructed by mapping the sequences of 383 pepper cultivars to the Zunla-1 genome as the reference [142]. The novel contig sequences (accession number GWHAAAT00000000) are available at http://bigd.big.ac.cn/gwh.
BRIDGEcereal Link: https://bridgecereal.scinet.usda.gov (accessed on 13 September 2023). Species: wheat, maize, barley, sorghum, and rice.	The Blastn Recovered Insertion and Deletion near Gene Explorer (BRIDGEcereal) web application supports mining publicly accessible pan-genomes of five major cereal crops, including wheat, maize, barley, sorghum, and rice [143]. It facilitates the identification of potential indels (insertion or deletions) for genes of interest.
PanSoy Link: https://www.soybase.org/projects/SoyBase.C2021.01.php (accessed on 13 September 2023). Species: *Glycine soja* (wild soybean) and *Glycine max* (soybean).	PanSoy is a soybean pan-genome assembly consisting of the genome sequence data from 204 phylogenetically and geographically distinct soybean accessions (GmHapMap collection). It was built using the de novo genome assembly method [144].
Sunflower Genome Database Link: https://www.sunflowergenome.org (accessed on 13 September 2023). Species: *Helianthus annuus* (sunflower).	Sunflower pan-genome was generated using sequence from 287 cultivated lines, 17 Native American landraces, and 189 wild accessions representing 11 compatible wild species. Raw data used for pan-genome construction is available at NCBI, and SNP data is available at the Sunflower Genome Database [145].
COTTONOMICS Link: http://cotton.zju.edu.cn (accessed on 13 September 2023). Species: cotton.	It provides genome-wide, gene-scale structural variations detected from 11 assembled allopolyploid cotton genomes and is linked to important agronomic traits [146].
BGH Link: https://banana-genome-hub.southgreen.fr (accessed on 13 September 2023). Species: *Musa Ensete,* and genomics data of 15 Musaceae species.	The Banana Genome Hub (BGH), a web-based platform, supports users in exploring genes and gene families, gene expression patterns, associated SNP markers, etc. Users can also view chromosome structures, synteny, presence, absence variation, and genome ancestry mosaics [99].
CPBD Links: http://citrus.hzau.edu.cn/ (accessed on 13 September 2023). Species: sweet orange (*Citrus sinensis*), mandarin (*Citrus reticulata*), pummelo (*Citrus grandis*), grapefruit (*Citrus paradisi*), and lemon (*Citrus limon*).	The Citrus Pan-genome to Breeding Database (CPBD) was built using 23 genomes of 17 citrus species and has genetic variation data from 167 citrus accessions mapped to two reference genomes [147].
CitGVD Links: http://citgvd.cric.cn/home/index (accessed on 13 September 2023). Species: citrus accessions.	The Citrus Genome Database (CitGVD) hosts genomic data, genetic variation data, and built-in analysis tools. It contains 1493258964 non-redundant SNPs, INDELs, and 84 phenotypes from 346 citrus individuals. Users can browse/search annotated genetic variations and visualize results graphically in a genome browser or tabular outputs [148].
Apple pan-genome Link: http://bioinfo.bti.cornell.edu/apple_genome (accessed on 13 September 2023). Species: apple (*Malus domestica*) and its wild progenitors *M. sieversii* and *M. sylvestris*.	Apple pan-genome was constructed using phased diploid genome assemblies of *Malus domestica* cv. Gala, *M. sieversii*, and *M. sylvestris*, and 91 sequenced genomes of additional accessions [149].
BnPIR Link: http://cbi.hzau.edu.cn/bnapus (accessed on 13 September 2023). Species: *Brassica oleracea*, *Brassica macrocarpa* (cultivated and wild cabbage), *Brassica napus.*	The Brassica napus pan-genome information resource (BnPIR) hosts eight high-quality *B. napus* reference genomes generated using PacBio sequencing and re-sequencing data from 1688 rapeseed accessions. It provides a pan-gene module, pan-genome Browser, and synteny data. It also hosts multi-omics data and common bioinformatics tools [150].
Cassava pan-genome Link: https://cassavabase.org/ (accessed on 13 September 2023). Species: cassava (*Manihot esculenta*).	Two high-quality, chromosome-scale haploid genome assemblies for African cassava cultivar TME204 (resistant to cassava mosaic diseases caused by African cassava mosaic viruses) were generated using a combination of short-read and long-read sequencing methods (Illumina PE reads, PacBio CLRs, and HiFi reads [151].
Other public pan-genome data available (not yet included in crop databases or supported by Genome Browser and associated tools)	
Pearl millet Link: http://117.78.45.2:91/home (accessed on 13 September 2023). Species: pearl millet.	Pearl millet pan-genome was constructed using whole genome assemblies of 11 accessions generated using a combination of PacBio long-read sequences, Bionano optical mapping data, Hi-C data, and Illumina short-read sequence data [55].
Sorghum pan-genome Link: The bulk data is available at http://dataverse.icrisat.org/dataset.xhtml?persistentId=doi:10.21421/D2/RIO2QM (accessed on 13 September 2023). Species: sorghum.	This pan-genome was assembled using iterative mapping of whole-genome sequence data from 176 sorghum accessions to a sorghum reference assembly v3.0.1 from Phytozome [152]. It has 209935 assembled contig sequences from 176 sorghum accessions. This data represent 35,719 genes (including 34,211 genes from reference).
Barley pan-genome Link: https://bitbucket.org/ipk_dg_public/barley_pangenome/src/master/ (accessed on 13 September 2023). https://galaxy-web.ipk-gatersleben.de/libraries (accessed on 13 September 2023). Species: barley cultivars and a wild relative.	This first-generation barley pan-genome consists of chromosome-scale sequence assemblies for the 20 barley varieties (including landraces, cultivars, and wild barley from global barley diversity collection) and whole-genome shotgun sequencing data from additional 300 barley accessions [11].
Soybean pan-genome The genetic diversity data is available at https://figshare.com/s/689ae685ad2c368f2568 (accessed on 13 September 2023). SNPs and small indels data from the 2,898 accessions are available at (http://bigd.big.ac.cn/gvm/getProjectDetail?project=GVM000063 (accessed on 13 September 2023). Species: *Glycine soja* (wild soybean), *Glycine max* (soybean).	This graph-based pan-genome assembly was generated using de novo genome assemblies of 26 representative soybean accessions [14]. The sequencing data, assembled chromosomes, unplaced scaffolds, and annotations from this project are available at the Genome Sequence Archive and Genome Warehouse database in BIG Data Center (https://bigd.big.ac.cn/gsa/index.jsp) under Accession Number PRJCA002030.
Chickpea pan-genome Links: Pan-genome assembly and annotations: https://doi.org/10.6084/m9.figshare.16592819 (accessed on 13 September 2023). The variant calls: https://cegresources.icrisat.org/cicerseq (accessed on 13 September 2023).	The chickpea pan-genome consists of genome sequence data from 3366 chickpea lines (including 3171 cultivated and 195 wild accessions) [153]. Additional data, including Manhattan and QQ-plots for Genome-Wide Association Study (GWAS) analysis, is available at https://doi.org/10.6084/m9.figshare.15015309.
Pigeon pea Link: https://research-repository.uwa.edu.au/en/datasets/pigeon-pea-pangenome-contig-assembly-annotation-snps-pav (accessed on 13 September 2023). Species: pigeon pea (*Cajanus cajan*).	The pigeon pea pan-genome consists of genome sequence data from 89 pigeon pea accessions, including 70 from South Asia, 8 from sub-Saharan Africa, 7 from South East Asia, 2 from Mesoamerica, and 1 from Europe. This pan-genome was generated using the reference genome assembly (C. cajan_V1.0) and iterative mapping and assembly method [154].
Sesame pan-genome Species: sesame (*Sesamum indicum* L.).	The sesame pan-genome was constructed by mapping genome sequence data from two landraces, *S. indicum* cv. Baizhima and Mishuozhima and two cultivars, Yuzhi11 and Swetha, to the *S. indicum* var. Zhongzhi13 reference genome [155].
Cotton Variome Links: Genetic variation is available at https://www.ncbi.nlm.nih.gov/bioproject/PRJNA576032 and https://figshare.com/s/cb3c104782a1dcd90ab0 (accessed on 13 September 2023). Species: *Gossypium hirsutum* and *Gossypium barbadense.*	Cotton Variome provides genetic variation data from 1961 cotton accessions [156].
Melon pan-genome Link: https://figshare.com/articles/dataset/melon_pangenome/17195072 (accessed on 13 September 2023).	Pan-genome of *Cucumismelo* L. consists of genome sequence data from 297 accessions [157].
Cucumber pan-genome Data availability: Genome assemblies of the 11 cucumber accessions have been deposited in NCBI GenBank under the accession number PRJNA657438.	The cucumber pan-genome graph was constructed using genome sequence data from 11 representative accessions from the 115-line core collection. The genome assemblies were generated using long-read and short-read sequence data [158].
Strawberry pan-genome The genome assembly and annotation files are available in the Genome Database for Rosaceae. The pan-genome browser or query support is not available. Link: https://www.rosaceae.org/species/fragaria/all (accessed on 13 September 2023). Species: cultivated and wild strawberry.	This strawberry pan-genome was generated using chromosome-scale reference genome assemblies of five diploid strawberry species (*Fragaria mandschurica*, *Fragaria daltoniana*, *Fragaria pentaphylla*, *F. nilgerrensis*, and *F. viridis*) and genome resequencing data of 128 accessions [159].
Walnut pan-genome Link:https://db.cngb.org/search/project/CNP0001209 (accessed on 13 September 2023). Species: walnut (*Juglans nigra*).	A high-quality reference genome assembly of black walnut (*Juglans nigra*) genotype NWAFU168 was constructed using short-read and long-read sequence data (Illumina, Pacbio, and Hi-C). A Walnut pan-genome was built using this reference genome and mapping sequence data from 74 walnut accessions [56].
SalviaGDB Link: https://salviagdb.org/ (accessed on 13 September 2023). Species: *Salvia hispanica* (Chia), *S. miltiorrhiza* (Danshen), *S. bowleyana* (nan Denshen)*, S. splendens* (sage), and *S. rosmarinus* (rosemary).	The high-quality genome assembly and annotations of orphan crop *Salvia hispanica* (Chia) (4 genomes), and one each for the herbs used in culinary and traditional medicine.

## Data Availability

We have provided all the data within this manuscript.
